# Protocol for genome-wide differential methylation using long-read DNA sequencing in the cortex of a mouse model of Alzheimer’s disease

**DOI:** 10.1016/j.xpro.2026.104827

**Published:** 2026-10-08

**Authors:** Ümran Yaman, Emil Gustavsson, Jasmaine Lee, Hermione Ugolini, Hannah Macpherson, Paige Mumford, Frances Wiseman, John Hardy, Dervis A. Salih

**Affiliations:** 1 UK Dementia Research Institute at UCL, Gower Street, London WC1E 6BT, UK; 2 Department of Neuromuscular Disease, UCL Queen Square Institute of Neurology, London WC1N 3BG, UK; 3 Department of Cognitive Neuroscience, Institute of Neurological Sciences, İstanbul-Cerrahpaşa University, İstanbul 34098, Türkiye; 4 Department of Neurodegenerative Disease, Queen Square Institute of Neurology, University College London, London WC1N 3BG, UK; 5 UK Dementia Research Institute at University of Cambridge, Cambridge CB2 0AH, UK; 6 Department of Clinical Neurosciences, School of Clinical Medicine, University of Cambridge, Cambridge CB2 0QQ, UK; 7 Department of Genetics and Genomic Medicine, Great Ormond Street Institute of Child Health, University College London, London WC1N 1EH, UK; 8 NIHR Great Ormond Street Hospital Biomedical Research Centre, University College London, London WC1N 1EH, UK

**Keywords:** Bioinformatics, Sequence analysis, Genomics, Immunology, Neuroscience, NMGN Focused Collection

## Abstract

Third-generation sequencing enables direct detection of genome-wide 5-methylcytosine without bisulfite conversion. Here, we present a protocol for generating long-read DNA sequencing from mouse cortex and performing genome-wide differential methylation analysis. We describe steps for high-molecular-weight DNA extraction, data generation, and downstream analysis. We demonstrate the workflow in the *App*^*NL-G-F*^ mouse model of amyloid pathology and provide a reproducible pipeline that links modified-base BAM (modBAM) files to annotated differentially methylated regions and functional enrichment results.

## Highlights

• Workflow for genome-wide methylation analysis using Oxford Nanopore Technologies DNA sequ
• Steps for covariate-aware differential methylation modeling using DSS
• Annotation and functional enrichment of differentially methylated regions
• Instructions for a reproducible Snakemake pipeline from modBAM to interpretable results

## Abstract (editor highlights)

Publisher’s note: Undertaking any experimental protocol requires adherence to local institutional guidelines for laboratory safety and ethics.

## Graphical abstract

**Figure:**
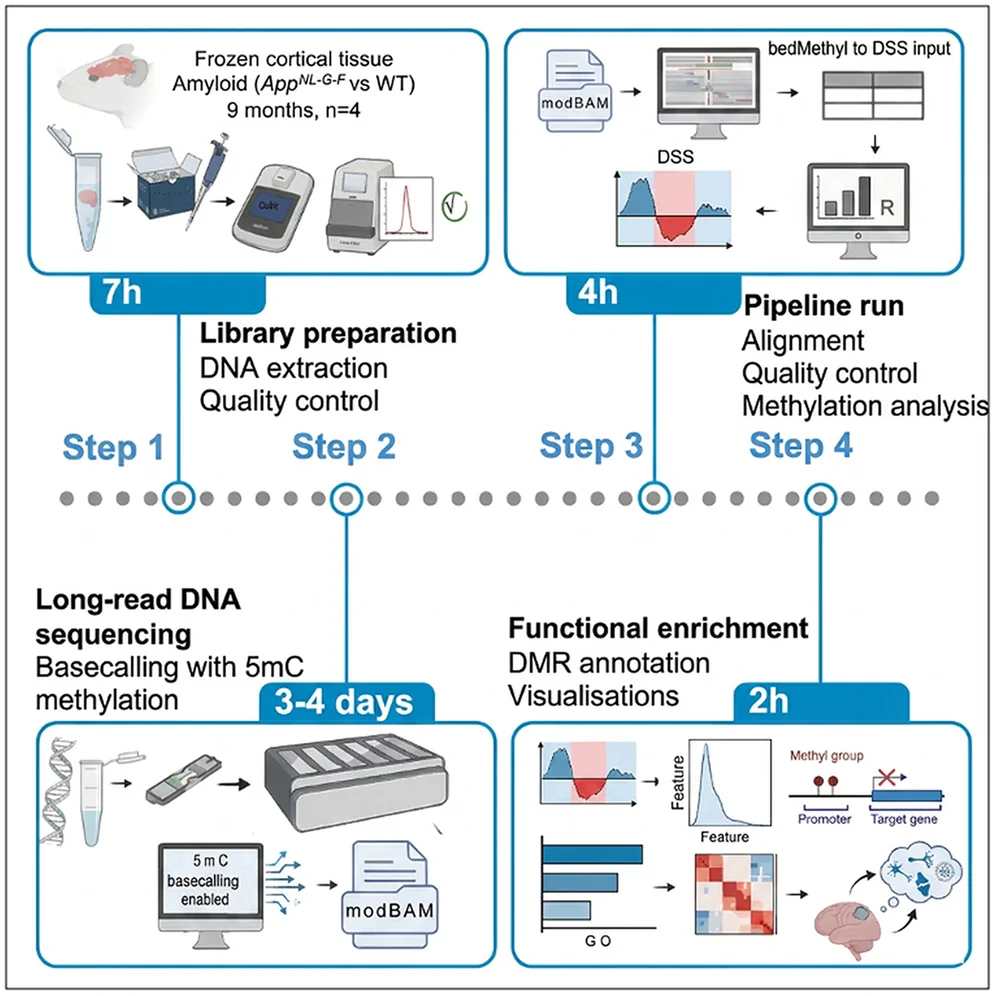

## Before you begin

### Innovation statement

This new protocol integrates the entire experiment from wet-laboratory and computational analysis procedures and codes for genome-wide differential methylation analysis using Oxford Nanopore native DNA sequencing, including biological interpretation with rGREAT^1^ and simplifyGO.^2^ While library preparation and base modification detection using Nanopore sequencing have been described previously,^3^ comprehensive workflows that guide users from raw sequencing outputs to statistically robust identification of differentially methylated regions remain limited.

The protocol combines long-read sequencing with covariate-aware statistical modeling using Dispersion Shrinkage for Sequencing (DSS) to detect differential methylation across multiple samples while accounting for biological and technical variables such as sex and sequencing batch. In addition, practical recommendations are included for high-molecular-weight (HMW) DNA handling and sequencing strategies to improve read length and methylation detection accuracy.

The workflow connects aligned modBAM files to biologically interpretable results through automated processing, genomic annotation, functional enrichment analysis, and visualization. The pipeline is implemented as a reproducible Snakemake workflow and R project that can be adapted to different experimental designs and datasets.

### Institutional permissions

All animals were co-housed and maintained at the Mary Lyon Centre, MRC Harwell Institute, under specific pathogen-free conditions in individually ventilated cages according to the Home Office Code of Practice. All animal procedures were conducted under a UK Home Office license in accordance with the Animals (Scientific Procedures) Act 1986 Amendment Regulations 2012 (SI 2012/3039) and approved by institutional ethical review committees.

DNA methylation, particularly 5-methylcytosine (5mC), plays an important role in regulating gene expression and chromatin state across the genome.^4^^,^^5^ Conventional genome-wide DNA methylation profiling has primarily relied on bisulfite conversion–based sequencing approaches,^6^^,^^7^^,^^8^ microarray-based platforms targeting predefined CpG loci such as Illumina Infinium arrays,^9^^,^^10^ and enrichment-based or enzyme-dependent methods for methylation detection.^11^^,^^12^ These approaches have several limitations, including DNA degradation during bisulfite treatment, incomplete conversion artifacts, restricted CpG coverage, and reduced mappability in repetitive or structurally complex genomic regions. Long-read sequencing technologies enable direct detection of 5mC from native DNA molecules without bisulfite conversion or PCR, preserving long-range genomic context and improving accessibility to repetitive and structurally complex loci.^13^^,^^14^^,^^15^

Alzheimer’s disease (AD) is a complex neurodegenerative disorder influenced by both genetic and epigenetic mechanisms that contribute to disease susceptibility and progression. Genome-wide association studies have identified numerous AD risk loci, yet the molecular mechanisms linking these loci to disease pathology remain incompletely understood.^16^^,^^17^^,^^18^^,^^19^ Increasing evidence suggests that epigenetic regulation, including DNA methylation and chromatin-associated factors, contributes to gene regulatory changes in the aging brain and in AD.^20^^,^^21^^,^^22^^,^^23^

This protocol provides an end-to-end workflow for genome-wide identification of differentially methylated loci (DMLs) and differentially methylated regions (DMRs) using Oxford Nanopore Technologies (ONT) long-read sequencing data. The workflow was developed using cortical genomic DNA from the *App*^*NL-G-F*^ knock-in mouse model of amyloid-β pathology^24^^,^^25^ compared with C57BL/6J wild-type controls (n=4 per group). Individual DMLs represent single CpG sites showing differential methylation but can be noisy and difficult to interpret biologically. Therefore, clustering neighboring DMLs into DMRs improves robustness and biological interpretability and is commonly used as the primary reporting unit in genome-wide methylation studies.^26^

The computational workflow begins with aligned modBAM files, which store base modification information generated during ONT basecalling. The analysis includes covariate-aware differential methylation modeling using DSS^27^^,^^28^^,^^29^^,^^30^ followed by DMR detection, genomic annotation, functional enrichment analysis, and visualization of representative loci. The protocol is designed as a hands-on guide for researchers new to the long-read DNA pipeline, linking generating raw sequencing outputs for genome-wide methylation analysis to biologically interpretable results through a reproducible workflow that can be adapted to different experimental designs and datasets.

## Key resources table

**Table:** 

REAGENT or RESOURCE	SOURCE	IDENTIFIER
**Experimental models: Organisms/strains**		

*App*^*NL-G-F*^ mice Species: Mus musculus Genotype: homozygous *App*^*NL-G-F/NL-G-F*^ knock-in. Age: 9 months. Sex: female and male	MRC Harwell colony – rederived from Riken BRC	*App*^*tm3.1Tcs*^ (here called *App*^*NL-G-F*^) MGI: 5637817
C57BL/6J Species: Mus musculus Genotype: wild-type. Age: 9 months. Sex: female and male	MRC Harwell colony	N/A

**Critical commercial assays**		

Monarch Genomic DNA Purification Kit	New England Biolabs	NEB#T3010
Ligation Sequencing Kit	Oxford Nanopore Technologies	SQK-LSK109
Native Barcoding Expansion (1–12)	Oxford Nanopore Technologies	EXP-NBD104
Native Barcoding Expansion (13–24)	Oxford Nanopore Technologies	EXP-NBD114
PromethION Flow Cell (R9.4.1)	Oxford Nanopore Technologies	FLO-PRO002

**Software and algorithms**		

MinKNOW	Oxford Nanopore Technologies	v23.04.6
MinKNOW core	Oxford Nanopore Technologies	v5.5.5
Bream	Oxford Nanopore Technologies	v7.5.10
Guppy Basecaller	Oxford Nanopore Technologies	v6.5.7 (HAC 450 bp; Q≥9; 5mC model enabled)
minimap2_c2.17-r941	Li et al.^31^	https://github.com/lh3/minimap2
SAMtools_v1.12	N/A	https://github.com/samtools/htslib
modkit_v0.6.1	N/A	https://github.com/nanoporetech/modkit
RStudio	Posit	v2026.04.0-526
R 4.1.1	R Development Core Team, 2021	https://www.R-project.org/
Snakemake	Mölder et al.^32^	https://github.com/snakemake/snakemake
rtracklayer_v1.62.0	Lawrence et al.^33^	https://bioconductor.org/packages/release/bioc/html/rtracklayer.html
DSS_v2.50.1	Wu et al.^28^	https://bioconductor.org/packages/release/bioc/html/DSS.html
bsseq_v1.38.0	Hansen et al.^34^	https://www.bioconductor.org/packages/release/bioc/html/bsseq.html
comethyl_v1.3.0	Mordaunt et al.^35^	https://github.com/cemordaunt/comethyl
rGREAT_v2.4.0	Gu and Huebschmann^1^	https://bioconductor.org/packages/rGREAT
simplifyEnrichment_v1.12.0	Gu and Huebschmann^2^	https://bioconductor.org/packages/simplifyEnrichment
GenomicRanges_v1.54.1	Lawrence et al.^33^	https://bioconductor.org/packages/release/bioc/html/GenomicRanges.html
GenomicFeatures_v1.54.4	Lawrence et al.^33^	https://bioconductor.org/packages/release/bioc/html/GenomicFeatures.html
Rsamtools_v2.18.0	Morgan et al.^36^	https://bioconductor.org/packages/Rsamtools
ggplot2_v4.0.2	Wickham^37^	https://github.com/tidyverse/ggplot2
dplyr_v1.2.0	Wickham et al.^38^	https://github.com/tidyverse/dplyr
ONT_DNA_pipeline_v1.0.0	This paper	https://github.com/umranyaman/ONT_DNA_pipeline
Original codes used in this manuscript	This paper	https://zenodo.org/records/20083920

**Deposited data**		

*Mus musculus* reference genome (mm10)	UCSC Genome Browser	https://genome.ucsc.edu
TxDb.Mmusculus.UCSC.mm10.knownGene_v3.10.0	UCSC Genome Browser	https://bioconductor.org/packages/release/data/annotation/html/TxDb.Mmusculus.UCSC.mm10.knownGene.html

Compute guidance: Linux workstation or HPC; ≥16 threads recommended; ≥64 GB RAM useful for genome-wide analysis.

## Materials and equipment

No custom-prepared recipes and buffers with more than two ingredients were used in this protocol; all reagents and buffers are components of commercial kits listed in the key resources table, prepared accordingly to the manufacturers’ instructions unless otherwise noted in the method details.

## Step-by-step method details

### Genotype animals and define the experimental design

**Timing: 4–6 h hands-on; 1–2 days total including qPCR scheduling**

1. Generate homozygous *App*^*NL-G-F*/*NL-G-F*^ and WT controls by heterozygous crosses maintained on a C57BL/6J background.
2. Collect ear biopsy tissue for genotyping.
3. Extract DNA from ear tissue using TaqMan Sample-to-SNP (Applied Biosystems) reagent or equivalent.
4. Perform multiplexed TaqMan qPCR genotyping for *App*^*NL*^ (amyloid-causal) and *App+* (WT) alleles with *Dot1l*/Zas reference using your validated primer/probe sets (See Table S1).
5. Record genotype calls and assign animals to groups balanced by sex and sequencing pool.

***Note:*** Alternative genotyping strategies are available.^24^^,^^25^ Commercial genotyping also an option via Transnetyx (Cordova, TN, USA) genotyping services.

### Animal husbandry, cortex dissection, and experimental design

**Timing: Variable (breeding); 30–45 min per animal for dissection and freezing**

6. Co-house mice by genotype and sex in individually ventilated cages under specific-pathogen-free conditions.

***Note:*** Avoid single housing where possible. Social isolation can modify amyloid-β–associated phenotypes.

7. Euthanize mice at 9 months by terminal perfusion with PBS under sodium pentobarbitone anesthesia according to institutional guidance.
8. Remove the whole brain, bisect along the midline, and dissect the cerebral cortex from one hemisphere (Figure S1).^39^
9. Flash-freeze cortical tissue immediately (e.g., liquid nitrogen) and store at −80°C until extraction.

**Pause point:** Store frozen cortex at −80°C for months before DNA extraction.

### Extract high-molecular-weight genomic DNA from cortex

**Timing: 4–5 h per 8 samples**

10. Keep tissue frozen and place on a chilled surface.
11. Weigh around 12 mg of frozen cortex (consistent across samples when possible).
12. Homogenize tissue according to the Monarch Genomic DNA Purification Kit (NEB T3010), following the manufacturer’s protocol with the modifications below to preserve DNA fragment length.

***Note:*** For sample homogenisation, grind frozen tissue with pestle while still frozen and add lysis buffer as soon as tissue becomes soft.

***Note:*** As an alternative to the Monarch kit, Qiagen or Promega HMW DNA extraction kits can be used.

13. For maximum DNA length, incubate samples at 56°C for 1.5 h. Shake samples for first 15 min, at 700 rpm.
14. After the incubation, add 10 μL RNAse A, invert samples 10 times, then incubate the samples at 56°C with an agitation speed of 700 rpm for 10 min.

**CRITICAL:** After the 10 min incubation, chill the lysate on ice for 3 min, before adding the protein separation solution for brain samples specifically.

15. Add 300 μL Protein Separation solution, and mix by inverting for 1 minute, centrifuge for 30 min at 16,000*g*. Transfer supernatant to the Monarch DNA-binding column.

**CRITICAL:** Using a 1000 μl wide-bore pipette tip, transfer the upper phase containing the DNA (large, clear phase, approx. 800 μl total volume) to a fresh tube. If a lower protein phase is not visible, leave ∼30 μl behind to ensure protein is not carried over.

16. Elute DNA in 100 μL Monarch elution buffer.
17. Store DNA at 4°C short-term (days) or −20°C long-term (weeks to months).
18. Quantify DNA using Qubit dsDNA HS.
19. Assess fragment length using Femto Pulse or equivalent.
20. Shear 3 μg DNA to ∼20 kb using a Megaruptor (or equivalent) to standardise read-length distributions.
21. Proceed only with high-integrity DNA suitable for long-read sequencing. The SQK-LSK109 ligation sequencing kit requires at least 1 μg (100–200 fmol) of sheared DNA.

**CRITICAL:** DNA integrity and input mass strongly affect sequencing yield and read length, where 50% of total bases are in reads of that length or longer (N50). Fragmented DNA reduces N50 and sequencing performance. Avoid repeated freeze–thaw cycles, vortexing, and excessive pipetting to preserve high-molecular-weight DNA. Optimal DNA integrity is suggested where >70% of sample fragments are >10 kb.

**CRITICAL:** Tapestation or Bioanalyzer can be used to assess DNA fragment size distribution; however, these platforms have limited resolution for HMW DNA and are generally unable to evaluate fragments above certain size thresholds accurately. Femto Pulse is recommended as it provides more reliable sizing and distribution analysis for large DNA fragments typically required for long-read sequencing.

### Prepare ONT native barcoded ligation libraries

**Timing: 1.5–3 h per sample**

22. Start with at least 1 μg genomic DNA per sample (or the ONT-recommended input for your kit version).
23. Prepare native barcoded ligation libraries using SQK-LSK109 with EXP-NBD104/EXP-NBD114 following the ONT protocol for native barcoding and ligation sequencing. Briefly, DNA is subjected to end-repair and dA-tailing, followed by native barcode ligation, clean-up, adapter ligation, and final library purification before sequencing.
24. Pool barcoded libraries so each pool includes samples from each genotype and sex where possible to reduce potential sequencing batch effects and improve experimental balance across flow cells.

**CRITICAL:** Balance genotype and sex across sequencing pools/flow cells to reduce confounding between biological and technical batch effects.

**CRITICAL:** Alternatives to the SQK-LSK109 (ONT) library preparation are the updated LSK114 (ONT) preparation or the SMRTbell preparation kit 3.0 (Pacific Biosciences).

### Run PromethION sequencing and perform 5mC-enabled basecalling

**Timing: ∼72–96 h per PromethION flow cell run**

25. Prepare your R9.4.1 PromethION flow cell (FLO-PRO002) according to ONT instructions.
26. Load the pooled library and start the run in MinKNOW (e.g., v23.04.6; Core v5.5.5; Bream v7.5.10).
27. Configure the run as needed (example: 96-h limit; active channel selection enabled; pore scans every 1.5 h).
28. Basecall using Guppy (e.g., v6.5.7) with the HAC 450 bp model and enforce Q≥9.
29. Enable modified-base calling for 5mC so basecalling outputs include MM/ML tags in BAM.
30. Save outputs (FAST5/FASTQ and unaligned modBAM) with clear sample/pool identifiers.

***Note:*** PacBio HiFi sequencing using SPRQ chemistry with 8M SMRT Cells on the Revio platform may be used as an alternative.

***Note:*** Sequencing time varies depending on sequencing yield and pore performance; this study used 4 flow cells with up to 8 samples distributed across runs.

## Quantification and statistical analysis

### Read alignment and methylation extraction

**Timing: 1 h/sample**

The following data analysis has been tested in Linux and HPC environments, and depending on the dataset size, it is recommended to have at least 32GB RAM, and 16 CPU cores.

1. Merge and sort multiple modBAM files if needed, using samtools (https://github.com/samtools/samtools).

***Note:*** This step is required only if modBAM files are provided per barcode and are located in a directory named barcodeX, where X corresponds to the barcode number used during sequencing (e.g. barcode01, barcode02, …). Each barcodeX directory is assumed to contain one or more modBAM files generated during basecalling with modified-base detection enabled.

# Merge + realign per-barcode modBAMs to produce an aligned, sorted, indexed modBAM per barcode.

# Assumption: input modBAMs for a given barcode are in a folder named “barcodeX/" (e.g., barcode09/).

# Replace X with the barcode number you are processing, and set REF to your reference fasta.

> X="09″

> BARCODE_DIR="barcode${X}" # e.g., barcode09

>IN_GLOB="../../bam_pass_PoolX_MethylX/${BARCODE_DIR}/∗.bam”

> REF="/path/to/reference.fa” # Absolute path for reference

> THREADS=50 # Based on your computational resource available

*# Work in a per-barcode output folder*

> mkdir -p "${BARCODE_DIR}_merged"

> cd "${BARCODE_DIR}_merged"

*# 1) Merge all BAMs for this barcode into a single BAM*

> samtools merge -@ "${THREADS}" -f “merged_${X}.bam” ${IN_GLOB}

*# 2) Sort + index merged BAM*

> samtools sort -@ "${THREADS}" -o “merged_sorted_${X}.bam” “merged_${X}.bam"

> samtools index -@ "${THREADS}" “merged_sorted_${X}.bam"

2. Convert it to FASTQ, align the reads containing modification tags to the mouse reference genome (https://genome.ucsc.edu, GRCm38/mm10) using minimap2 (https://github.com/lh3/minimap2), then sort and index via samtools.

***Note:*** The goal of this step below is to produce one aligned, sorted, and indexed modBAM file per barcode, which can then be used as input for methylation extraction (e.g. via modkit) or for the downstream Snakemake pipeline.

*# 3) BAM to FASTQ (retain MM/ML tags) then align with minimap2*

> samtools fastq -@ "${THREADS}" -T MM, ML “merged_sorted_${X}.bam” \

 | minimap2 -ax map-ont -t "${THREADS}" -y "${REF}" - \

 | samtools view -@ "${THREADS}" -b -o “aligned_${X}.mod.bam” -

*# 4) Sort + index aligned modBAM* > samtools sort -@ "${THREADS}" -o “aligned_sorted_${X}.mod.bam” “aligned_${X}.mod.bam".

> samtools index -@ "${THREADS}" “aligned_sorted_${X}.mod.bam"

*# confirm MM/ML tags exist in output*

> samtools view -h “aligned_sorted_${X}.mod.bam” | head -n 200 | grep -E “MM:Z:|ML:B:C" -m 5 || \

 echo “WARNING: MM/ML tags not detected in first 200 lines. Check basecalling/tag retention."

**CRITICAL:** This study aligned to mm10 due to downstream tool compatibility. If aligned with mm39, genome-lifting may be required for annotation steps depending on the reference databases used.

**CRITICAL:** Confirm that modified-base tags persist through alignment and BAM processing. If tags are absent, regenerate modBAM using a modbase-enabled basecaller configuration.

3. Once you have obtained the aligned modBAM files, follow the steps in the snakemake pipeline available at github.com/umranyaman/ONT_DNA_pipeline for processing the aligned modBAM file and generating the outputs for the downstream analysis for the next step. Convert aligned modBAMs into bedMethyl files using modkit via pipeline and then convert bedMethyl outputs to TSV input for downstream R-based analysis.

### Setting up R environment

**Timing: 30 min**

4. R and RStudio should be installed before starting the downstream analysis. Navigate to the directory containing the R project downloaded from Zenodo (see key resources table).
5. Open the.Rproj file to launch the project in RStudio.
6. In the R console, install and activate the environment manager:

> install.packages(“renv”)

> library(renv)

> renv::activate() # Activate project-specific R environment

This step initialises the project environment and restores all required libraries for the downstream analysis defined in the R Markdown files.

7. To verify that all required packages are installed and up to date, run:

> renv::snapshot()

This command checks for missing or unsynchronised packages. If additional packages are required, follow the prompt and select the appropriate option e.g., selecting option 2, which loads any Bioconductor or CRAN libraries automatically and snapshots the current environment into environment lockfile (renv.lock). If all dependencies are already satisfied, the message “The lockfile is already up to date.” will be returned.

### Data import and initial preprocessing

**Timing: 1 h**

8. Import data into R for DSS-based analysis.

*> library(bsseq)*

*# Snakemake writes to:*

*# results/{Pool}/mod/5mC/{Genotype}/DSS/5mC.{Pool}_{Genotype}.dss.tsv*

> project_dir <- "." # run from workflow root

> out_root <- file.path(project_dir, “results")

> samplesheet <- “data/samplesheet.csv"

> meta <- read.csv(samplesheet, stringsAsFactors = FALSE, check.names = FALSE)

> meta$pool <- trimws(meta$pool)

> meta$genotype <- trimws(meta$genotype) # or your test condition

> meta$sex <- toupper(trimws(as.character(meta$sex))) #optional

> meta$batch <- trimws(as.character(meta$batch)) # optional

> MOD_TYPE <- “5mC".

*> meta$dss* <- file.path(out_root, meta$pool, “mod”, MOD_TYPE, meta$genotype, “DSS”, paste0(MOD_TYPE, ".", meta$pool, "_", meta$genotype, ".dss.tsv"))

*# Order: Pool1.Pool4 then WT before App^NL-G-F^*

> pool_levels <- paste0(“Pool”, 1:4) # change it based on your pool information

> meta$pool <- factor(meta$pool, levels = pool_levels)

*# Make genotype order, e.g., WT as a reference point*

> if (all(c(“WT","AppNLGF”) %in% unique(meta$genotype))) {

 meta$genotype <- factor(meta$genotype, levels = c(“WT","AppNLGF"))

}

> meta <- meta[order(meta$pool, meta$genotype), ]

> read_dss <- function(f) {

 d <- read.delim(f, sep = "\t", header = TRUE, stringsAsFactors = FALSE, check.names = FALSE)

 data.frame(

  chr = d$chr,

  pos = d$pos,

  M = d$X,

  Cov = d$N,

 stringsAsFactors = FALSE

 )

}

> dss_list <- lapply(meta$dss, read_dss)

*# Edit the design table below based on your experimental design*

> design <- data.frame(

 SampleID = ifelse(

  is.na(meta$sex) & is.na(meta$batch),

  paste(meta$pool, meta$genotype, sep = "_"),

  paste(meta$genotype, meta$sex, meta$batch, meta$pool, sep = "-")

 ),

 Strain = factor(as.character(meta$genotype), levels = c(“WT","AppNLGF")),

 Sex = factor(meta$sex, levels = c(“M","F")),

 Batch = factor(meta$batch),

 Pool = factor(as.character(meta$pool), levels = pool_levels),

 DSS = meta$dss,

 stringsAsFactors = FALSE

)

> print(design)

*# Build BSseq Object*

> BSobj <- makeBSseqData(dat = dss_list, sampleNames = design$SampleID)

*> pData(BSobj) <- design*

# Save the outputs

> out_dir <- file.path(project_dir, “results_R″)

> dir.create(out_dir, recursive = TRUE, showWarnings = FALSE)

> saveRDS(BSobj, file.path(out_dir, “BSobj_mm10.rds"))

> write.csv(design, file.path(out_dir, “samplesheet_design.csv”), row.names = FALSE)

BSobj

An object of type ‘BSseq’ with

 21862175 methylation loci

 8 samples

has not been smoothed

All assays are in-memory

### Filtering and quality control

**Timing: 20 min for Step 9, 5 min for Step 10 and 11 each**

Filtering was applied to retain CpG sites with sufficient coverage and variability using comethyl,^34^^,^^35^ with thresholds optimized using summary/diagnostic functions before final selection (Figure 1).

9. Filter CpG sites using comethyl::filterRegions() with minimum coverage threshold: covMin ≥ 10 reads per site in all samples, and methylation standard deviation threshold: methSD ≥ 0.05 (5%).

> library(comethyl)

> library(GenomicRanges)

> out_fig_dir <- file.path(project_dir, “results”, “figures")

> out_obj_dir <- file.path(project_dir, “results”, “objects”)

> dir.create(out_fig_dir, recursive = TRUE, showWarnings = FALSE)

> dir.create(out_obj_dir, recursive = TRUE, showWarnings = FALSE)

*# QC of the CpG sites, calculating CpG totals at specified cov and perSample cutoffs*

> CpGtotals <- getCpGtotals(

 BSobj,

 file = file.path(out_fig_dir, “CpG_Totals.txt")

)

> plotCpGtotals(

 CpGtotals,

 file = file.path(out_fig_dir, “CpG_Totals.pdf")

)

“cov” “perSample” “nSample” “nCpGs_M"   “perCpGs".

 0    0.5   4     21.862175     1

 0    0.55   5     21.862175     1

 0    0.6   5     21.862175     1

> BSobj_filtered <- filterCpGs(

 BSobj,

 cov = 2,

 perSample = 0.75,

 file = file.path(out_obj_dir, “BSseq_filterCpGs_cov2_per0.75.rds")

)

***Note:*** CpG sites were filtered to retain loci with ≥2 reads in at least 75% of samples, which preserved ∼97% of total CpGs while removing low-confidence low-coverage sites, optionally can be visualised by regional values.

[getCpGs] Final BSseq Object:

An object of type ‘BSseq’ with

 21295677 methylation loci

 8 samples

has not been smoothed

All assays are in-memory

10. Optimize cutoffs using, getRegionTotals(), plotRegionTotals() based on your dataset. getRegions() calculated region statistics, where region is defined as at least 3 CpGs are no more than 150 bases apart by default.

*# Define regions and optimise filtering*

> regions <- getRegions(

 BSobj,

 file = file.path(out_fig_dir, “Regions_unfiltered.txt")

)

***Note:*** Region-level statistics summarize genomic coordinates, CpG density, sequencing coverage depth, and methylation levels. Specifically, *width* reflects the physical size of the genomic region; *n* represents the number of CpG sites per region; *covMin*, *covMean*, and *covSD* describe the minimum, mean, and variability of sequencing coverage across CpGs within each region; and *methMean* and *methSD* capture the average methylation level and methylation variability across CpGs within the region.

**Figure 1:**
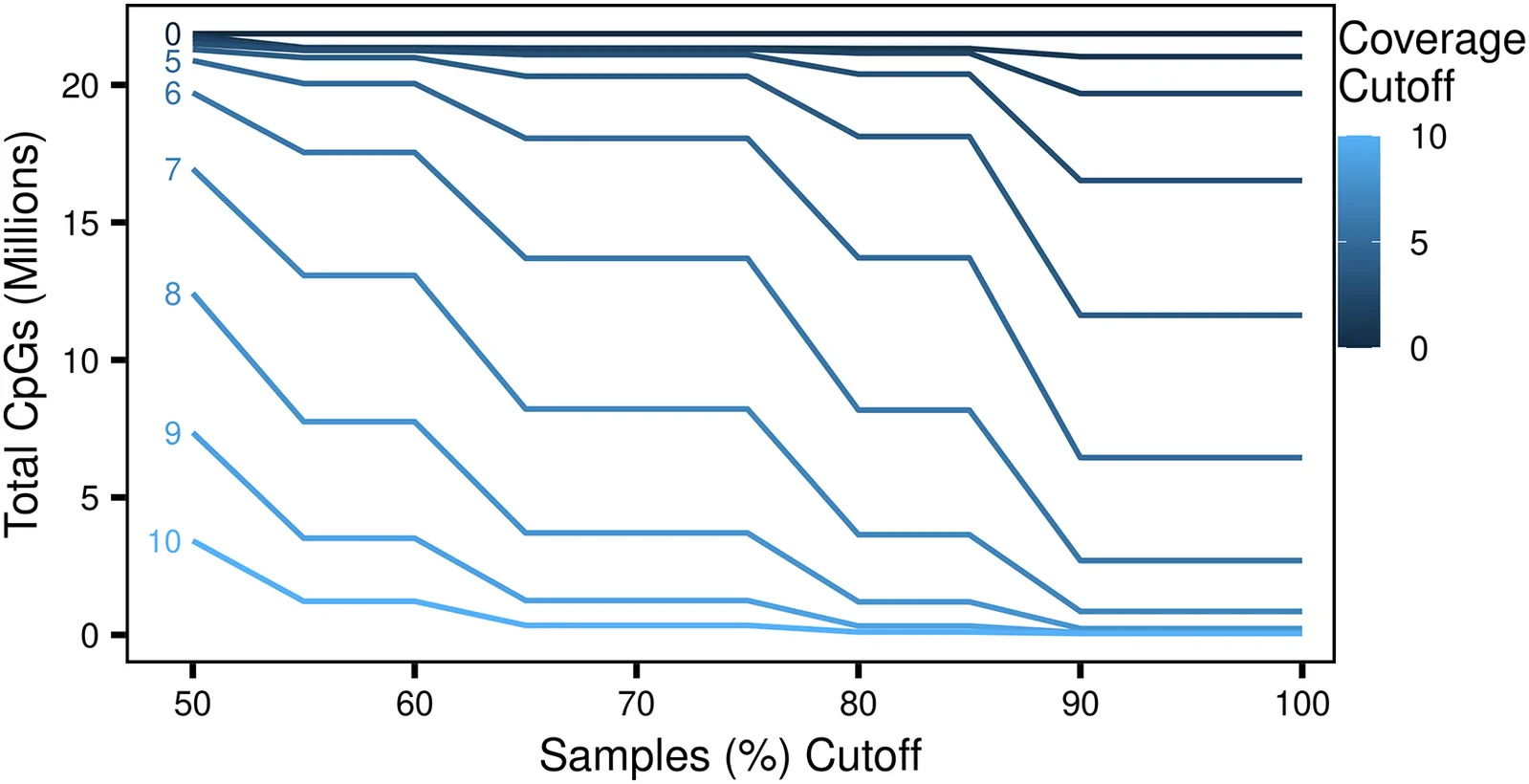
Total CpGs retained across filtering thresholds Total number of CpG sites (millions) retained as a function of the minimum percentage of samples required (x-axis) and coverage cutoff (color scale, cov = 0–10). Increasing either the coverage threshold or the required sample proportion progressively filters the number of retained CpGs.

In this dataset, regions were filtered to retain loci with minimum coverage (covMin) ≥10 reads, and methylation variability (methSD) ≥0.05. These thresholds correspond to the stability plateau where methylation variance becomes largely independent of sequencing coverage, while preserving the majority of informative genomic CpG regions for downstream analysis.

The dataset contains approximately 21.86 million CpG sites in total, and CpG retention depends on both minimum read coverage per sample (cov) and the fraction of samples required to meet that coverage (perSample); for example, when requiring at least 1 read in all 8 samples (cov = 1, perSample = 1), about 21.03 million CpGs are retained (∼96% of all CpGs), indicating very consistent coverage across samples, whereas increasing stringency reduces CpG retention, such that requiring ≥5 reads in all samples retains only about 6.45 million CpGs (∼29%), and requiring ≥10 reads in all samples retains only about 0.05 million CpGs (∼0.23%), demonstrating stricter coverage and completeness thresholds reduce the number of CpGs available for downstream methylation analysis.

# Filter regions based on the optimised thresholds

> regions_filt <- filterRegions(

 regions,

 covMin = 10,

 methSD = 0.05,

 file = file.path(out_fig_dir, “Regions_filtered_covMin10_methSD0.05.txt")

)

11. Re-examine retained regions as in step 10, and change thresholds if needed.

***Note:*** `filterRegions()` can return either a data.frame-like object or GRanges, depending on comethyl version/settings.

> if (inherits(regions_filt, “GRanges")) {

 gr_regions <- regions_filt

} else {

 # Expect columns: chr, start, end

 req_cols <- c(“chr”, “start”, “end")

  if (!all(req_cols %in% colnames(regions_filt))) {

   stop(

    “Filtered regions object does not contain expected columns: ",

    paste(req_cols, collapse = ", "),

    “\nFound: ", paste(colnames(regions_filt), collapse = ", ")

  )

 }

 gr_regions <- GRanges(

  seqnames = regions_filt$chr,

  ranges = IRanges(start = regions_filt$start, end = regions_filt$end)

 )

}

*# Subset the BSseq object using the filtered regions*

> BSobj_filtered_by_regions <- subsetByOverlaps(BSobj_filtered, gr_regions)

> message(“Number of CpGs after subsetting by filtered regions: ", nrow(BSobj_filtered_by_regions))

> saveRDS(

 BSobj_filtered_by_regions,

 file.path(out_obj_dir, “BSobj_filtered_by_regions.rds")

)

*# Optional: also save the GRanges for reuse*

> saveRDS(

 gr_regions,

 file.path(out_obj_dir, “filtered_regions_GRanges.rds")

)

After filtering by region, 1357100 regions, which corresponds to 8513584 loci remained in the BSobject.

### Differential methylation analysis

**Timing: 4 h**

DSS performs Wald tests at each CpG to test whether genotype is significantly associated with methylation relative to the reference level.^27^^,^^28^^,^^29^^,^^30^ Fit a multi-factor model using DMLfit.multiFactor() using DSS with covariates as follows: Genotype (also referred to as Strain), sex, and batch (sequencing pool/flow cell).

***Note:*** These are the covariates from the design matrix above and can be modified depending on the experimental design.

Model formula:

Methylation ∼ Genotype + Sex + Batch

Smoothing was applied (smoothing = TRUE) to leverage spatial correlation between neighboring CpGs and improve statistical power.

# Model design + DSS multi-factor fitting

# Match design rows to BSseq sample order

> colnames(BSobj_filtered_by_regions) <- BSobj_filtered_by_regions@colData@listData$SampleID

> rownames(design) <- design$SampleID

> rownames(design) == colnames(BSobj_filtered_by_regions) # Expect TRUE

*# Save the design used*

> write.csv(design, file.path(out_obj_dir, “design2.csv”), row.names = FALSE)

***Note:*** Before fitting multi-factor DSS models, verify that their experimental design matrix is full rank and has positive residual degrees of freedom, as insufficient replication or confounded covariates can lead to unstable or non-estimable model coefficients.

*# DSS model fitting*

> DMLfit <- DMLfit.multiFactor(

 BSobj_filtered_by_regions, design = design,

 formula = ∼ Strain + Sex + Batch,

 smoothing = TRUE

)

> saveRDS(DMLfit, file.path(out_obj_dir, “DMLfit_mm10.rds"))

*# Show DSS design matrix columns actually used*

> print(colnames(DMLfit$X))

[1] "(Intercept)" “StrainAppNLGF” “SexF” “BatchB” “BatchC” “BatchD"

*# Choose coefficient for genotype/strain effect, expecting something like* “StrainAppNLGF”, this will be different based on the namings of your experimental design

> DMLtest.Strain <- DMLtest.multiFactor(DMLfit, coef = “StrainAppNLGF")

> saveRDS(DMLtest.Strain, file.path(out_obj_dir, “DMLtest_Strain_mm10.rds"))

> ix <- sort(DMLtest.Strain[,"pvals"], index.return = TRUE)$ix

> head(DMLtest.Strain[ix, ])

Instead, we recommend proceeding with region-based analysis, which increases statistical power by aggregating signals across neighboring CpGs as below.

### Identification of differentially methylated regions

**Timing: 10 min**

12. Use callDMR() to aggregate single CpG test results, and find clusters of nearby significant CpGs, then merge them into DMRs.

> dmrs = callDMR(DMLtest.Strain, p.threshold=0.05)

***Note:*** callDMR() aggregates nearby CpGs with nominally significant methylation differences into DMRs and computes areaStat as a regional summary of consistent methylation change.

**Table:** 

> head(dmrs)						
	chr <chr>	start <dbl>	end <dbl>	length <dbl>	nCG <dbl>	areaStat <dbl>
6002	chr12	3235412	3236009	598	59	−118.19288
18602	chr2	116061381	116062065	685	35	79.35754
25831	chr6	4748102	4748493	392	37	77.73651
18630	chr2	118554994	118555470	477	36	76.77252
4754	chr11	51668675	51669189	515	29	−71.09874
13532	chr16	94205043	94205877	835	28	−69.92452

### Genomic annotation of DMRs

**Timing: 15 min**

13. Convert DMRs to a GRanges object. Load
  TxDb.Mmusculus.UCSC.mm10.knownGene for mm10 annotations

> library(GenomicRanges)

> library(IRanges)

> library(GenomicFeatures)

> library(GenomeInfoDb)

> library(AnnotationDbi)

> library(TxDb.Mmusculus.UCSC.mm10.knownGene)

> library(org.Mm.e.g.,.db)

> library(dplyr)

> library(rGREAT)

> library(simplifyEnrichment)

> out_tab_dir <- file.path(project_dir, “results_R″, “tables")

> dir.create(out_tab_dir, recursive = TRUE, showWarnings = FALSE)

> dmr_gr <- GRanges(

 seqnames = dmrs$chr,

 ranges = IRanges(start = dmrs$start, end = dmrs$end)

)

> txdb <- TxDb.Mmusculus.UCSC.mm10.knownGene

*# Make sure seqname style matches TxDb (mm10 TxDb is UCSC “chr1″ style)*

*# Make styles match first (UCSC chr1 style)*

> seqlevelsStyle(dmr_gr) <- “UCSC"

*# Keep only chromosomes that exist in both*

> common <- intersect(seqlevels(dmr_gr), seqlevels(txdb))

> dmr_gr <- keepSeqlevels(dmr_gr, common, pruning.mode = “coarse")

14. Perform the promoter overlap and annotate the nearest TSS with promoter definition: −2000 to +500 around TSS. For DMRs outside promoter regions, assign the nearest gene by distance to the nearest TSS.

> genes_gr <- genes(txdb) *# gene bodies (Entrez IDs stored as gene_id)*

> tss_gr <- resize(genes_gr, width = 1, fix = “start”) # TSS at gene start (TxDb already strand-aware)

> prom_gr <- promoters(genes_gr, upstream = 2000, downstream = 500)

*# promoter overlaps*

> hits_prom <- findOverlaps(dmr_gr, prom_gr, ignore.strand = TRUE)

> isPromoter <- rep(FALSE, length(dmr_gr))

> isPromoter[unique(queryHits(hits_prom))] <- TRUE

*# nearest TSS for all DMRs (for reporting and gene assignment for non-promoters)*

> nearest_tss <- distanceToNearest(dmr_gr, tss_gr, ignore.strand = TRUE)

> nearest_gene_id <- as.character(tss_gr$gene_id[subjectHits(nearest_tss)])

> nearest_dist <- mcols(nearest_tss)$distance

*# assign gene_id:*

*# - if promoter overlap: assign one overlapping promoter gene_id (first hit)*

*# - else: assign nearest TSS gene_id*

> gene_id <- nearest_gene_id

> if (length(hits_prom) > 0) {

* # choose first promoter gene per DMR*

 prom_gene <- tapply(as.character(prom_gr$gene_id[subjectHits(hits_prom)]),

       queryHits(hits_prom),

       function(x) x[1])

 gene_id[as.integer(names(prom_gene))] <- unname(prom_gene)

}

*# distanceToTSS: 0 for promoter overlaps, else nearest distance*

> distanceToTSS <- nearest_dist

> distanceToTSS[isPromoter] <- 0L

> annotation <- ifelse(isPromoter, “Promoter (-2000,+500)", “Distal (nearest TSS)")

*# map Entrez -> SYMBOL*

> SYMBOL <- AnnotationDbi::mapIds(

 org.Mm.e.g.,.db,

 keys = gene_id,

 column = “SYMBOL”,

 keytype = “ENTREZID”,

 multiVals = “first"

)

> dmrs_annot <- dmrs %>%

 mutate(

  annotation = annotation,

  gene_id = gene_id,

  SYMBOL = unname(SYMBOL),

  distanceToTSS = distanceToTSS

 )

> saveRDS(dmrs_annot, file.path(out_obj_dir, “dmrs_annotated_mm10.rds"))

> write.csv(dmrs_annot, file.path(out_tab_dir, “dmrs_annotated_mm10.csv”), row.names = FALSE)

> print(head(dmrs_annot[, c(“chr","start","end","areaStat","annotation","SYMBOL","distanceToTSS")], 5))

Distal (nearest TSS) Promoter (-2000,+500)

      2667          68

**CRITICAL:** Chromosome naming must match across BAM/FASTA/TxDb; mismatches are a frequent failure point.

*# areaStat positive values indicate hypermethylation and negative values indicate hypomethylation in App^NL-G-F^ relative to WT.*

> dmrs_annot <- dmrs_annot %>%

 mutate(

  Type = ifelse(areaStat < 0, “Hypomethylated”, “Hypermethylated"),

  isPromoter = annotation == “Promoter (-2000,+500)"

 )

### Functional enrichment analysis of DMRs

**Timing: 1 h**

15. Split DMRs by direction (hyper vs hypo) using areaStat for downstream analyses.

*# rGREAT enrichment (promoters vs all DMRs)*

> run_great_bp <- function(df, label) {

 if (nrow(df) < 10) {

  message(“Too few regions for GREAT: ", label)

  return(NULL)

 }

 gr <- GRanges(seqnames = df$chr,

     ranges = IRanges(df$start, df$end))

 seqlevelsStyle(gr) <- “UCSC".

 res <- rGREAT::great(gr,

       gene_sets = “GO:BP”,

       tss_source = “mm10″) # or

       “TxDb.Mmusculus.UCSC.mm10.knownGene".

tb <- rGREAT::getEnrichmentTable(res)

 write.csv(tb,

   file.path(out_tab_dir, paste0(“GREAT_", label, "_GO_BP_all.csv")),

   row.names = FALSE)

 tb_sig <- dplyr::filter(tb, p_value < 0.05)

 write.csv(tb_sig,

    file.path(out_tab_dir, paste0(“GREAT_", label, "_GO_BP_p_lt_0.05.csv")),

    row.names = FALSE)

 list(res = res, table = tb, table_sig = tb_sig)

}

16. Perform GO enrichment using rGREAT,^1^ focusing primarily on promoter-associated DMRs, with optional extension to all DMRs.

*# Run GREAT on promoter-only and all DMRs, split by direction*

> hyper_prom <- dmrs_annot %>% filter(Type == “Hypermethylated”, isPromoter)

> hypo_prom <- dmrs_annot %>% filter(Type == “Hypomethylated”, isPromoter)

> hyper_all <- dmrs_annot %>% filter(Type == “Hypermethylated")

hypo_all <- dmrs_annot %>% filter(Type == “Hypomethylated")

> res_hyper_prom <- run_great_bp(hyper_prom, “hypermethylated_promoters")

> res_hypo_prom <- run_great_bp(hypo_prom, “hypomethylated_promoters")

> res_hyper_all <- run_great_bp(hyper_all, “hypermethylated_allDMRs")

> res_hypo_all <- run_great_bp(hypo_all, “hypomethylated_allDMRs")

Most DMRs are expected to occur distal to transcription start sites, with relatively fewer in promoter regions. Consistently, in this dataset, DMRs were mainly distal (2667 distal vs 68 promoter). GREAT Gene Ontology-Biological Process (GO-BP) analysis of both hyper and hypomethylated promoters and genome-wide DMRs supported functional interpretation of methylation-linked regulatory changes.

17. Cluster enriched GO terms using simplifyEnrichment^2^ with the binary_cut method.

*# simplifyEnrichment: cluster significant GO IDs (p<0.05)*

> simplify_go <- function(tb, label) {

 if (is.null(tb) || nrow(tb) == 0) return(NULL)

 sig_ids <- tb$id[tb$p_value < 0.05]

 sig_ids <- sig_ids[!is.na(sig_ids)]

 if (length(sig_ids) < 5) {

  message(“Too few significant GO IDs for simplifyGO: ", label)

  return(NULL)

 }

 cl <- simplifyGO(sig_ids, method = “binary_cut")

 saveRDS(cl, file.path(out_obj_dir, paste0(“simplifyGO_", label, ".rds")))

 cl

}

> cl_hyper_prom <- simplify_go(res_hyper_prom$table, “hyper_promoters")

> cl_hypo_prom <- simplify_go(res_hypo_prom$table, “hypo_promoters")

> cl_hyper_all <- simplify_go(res_hyper_all$table, “hyper_all")

> cl_hypo_all <- simplify_go(res_hypo_all$table, “hypo_all")

***Note:*** This step should produce clusters of related GO:BP terms from the significant enrichment results, reducing redundancy and grouping similar biological themes. For each input set, the output is a simplifyGO clustering object (saved as.rds) containing GO term clusters that represent higher-level biological processes rather than many overlapping GO terms. If sufficiently significant GO IDs are present (≥∼5 terms with p < 0.05), you should obtain stable clusters highlighting dominant biological themes; otherwise, the function will return NULL and print a message indicating too few terms for reliable clustering.

18. Visualisation of GO-BP terms from hypomethylated promoter-associated DMRs as an example, simplifyGO cluster assignments were merged with the corresponding rGREAT enrichment results using GO term identifiers. Semantic similarity between GO terms was computed using GO_similarity() from the simplifyEnrichment package. For clarity, the top enriched GO terms per cluster (ranked by enrichment p-value) were selected and visualised as a similarity heatmap using ComplexHeatmap. GO terms were grouped by simplifyGO clusters to highlight higher-level biological themes while reducing redundancy among related terms (Figure 2).

**Figure 2:**
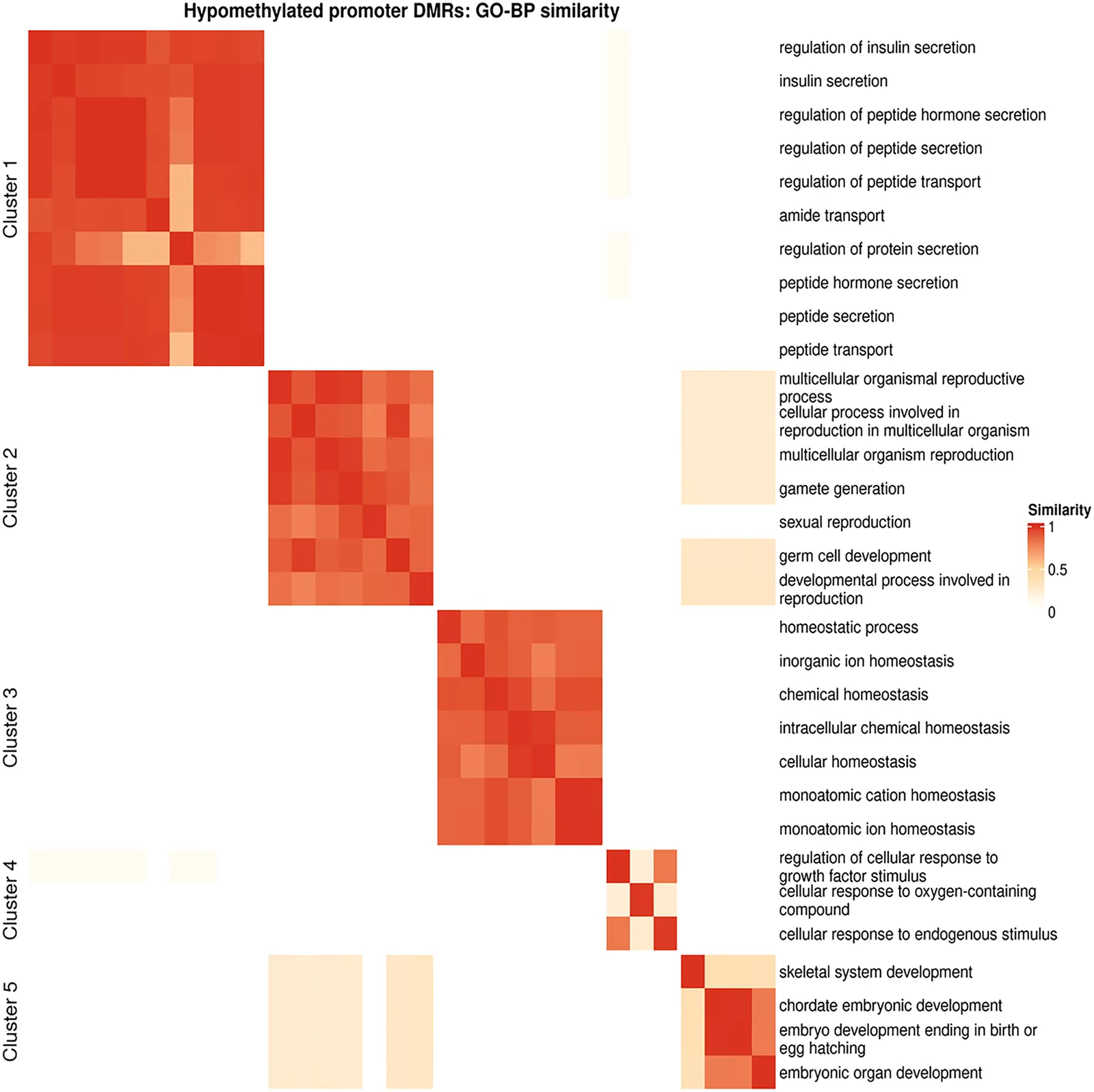
GO-BP similarity heatmap of hypomethylated promoter DMRs Enriched GO-BP terms from hypomethylated promoter-associated DMRs were clustered using simplifyEnrichment(binary_cut). The heatmap shows pairwise semantic similarity (0–1, white to red), with terms grouped into clusters representing major biological themes.

> library(stringr)

> library(ComplexHeatmap)

> library(circlize)

# Merge simplifyGO clusters with GREAT results

> cl_hypo_prom_df <- cl_hypo_prom %>%

 left_join(as.data.frame(res_hypo_prom$table), by = “id")

# Select top GO terms per cluster (based on p-value)

> df2 <- cl_hypo_prom_df %>%

 filter(!is.na(id), !is.na(description), !is.na(cluster), !is.na(p_value)) %>%

 arrange(cluster, p_value) %>%

 group_by(cluster) %>%

 slice_head(n = 10) %>%

 ungroup()

# Compute GO similarity matrix and order by id

> sim <- GO_similarity(df2$id, ont = “BP")

> sim <- sim[df2$id, df2$id]

# Labels

> row_labels <- stringr::str_wrap(df2$description, width = 40)

> ht <- Heatmap(

 sim,

 name = “Similarity",

 col = circlize::colorRamp2(c(0, 0.5, 1), c(“white”, "#FDD49E″, "#D7301F″)),

 cluster_rows = FALSE,

 cluster_columns = FALSE,

 show_row_names = TRUE,

 show_column_names = FALSE,

 row_labels = row_labels,

 row_split = factor(df2$cluster),

 column_split = factor(df2$cluster),

 row_title = “Cluster %s",

 column_title = “Hypomethylated promoter DMRs: GO-BP similarity",

 row_names_gp = grid::gpar(fontsize = 11),

 column_title_gp = grid::gpar(fontsize = 13, fontface = “bold")

)

> tiff(“results_R/figures/hypo_promoters_GO_similarity.tiff”,

  width = 11, height = 10, units = “in”, res = 300, compression = “lzw")

> draw(ht)

> dev.off()

### Visualization of methylation patterns

**Timing: 15 min**

19. Visualise the distances of genomic features such as exons, 5′ and 3′ UTRs, distal intergenic regions to the differentially hyper/hypomethylated sites.

> library(dplyr)

> library(ggplot2)

> dmrs_annot <- dmrs_annot %>%

 mutate(

  Type = ifelse(areaStat < 0, “Hypomethylated”, “Hypermethylated"),

  Main_Annotation = case_when(

   grepl(“Promoter”, annotation) ∼ “Promoter",

   grepl(“Exon”, annotation) ∼ “Exon",

   grepl(“Intron”, annotation) ∼ “Intron",

   grepl(“Distal Intergenic”, annotation) ∼ “Distal Intergenic",

   grepl(“3′ UTR”, annotation) ∼ “3′ UTR",

   grepl(“5′ UTR”, annotation) ∼ “5′ UTR",

   TRUE ∼ “Other".

  )

 )

> feature_dist <- dmrs_annot %>%

 count(Main_Annotation, Type) %>%

 mutate(Percentage = 100 ∗ n/sum(n))

> p_feat <- ggplot(feature_dist, aes(x = reorder(Main_Annotation, Percentage), y = Percentage, fill = Type)) +

 geom_col(position = “dodge”) +

 coord_flip() +

 labs(title = “DMR genomic feature distribution”, x = "", y = “Percentage (%)") +

 theme_minimal(base_size = 12)

> ggsave(file.path(out_fig_dir, “DMR_feature_distribution.pdf”), p_feat, width = 7, height = 5)

*# Distance-to-TSS bins*

> dmrs_tss <- dmrs_annot %>%

 mutate(DistanceCategory = case_when(

  distanceToTSS <= 1000 ∼ “0-1kb",

  distanceToTSS <= 2000 ∼ “1-2kb",

  distanceToTSS <= 5000 ∼ “2-5kb",

  distanceToTSS <= 10000 ∼ “5-10kb",

  distanceToTSS <= 100000 ∼ “10-100kb”,

  TRUE ∼ ">100kb".

 )) %>%

 count(Type, DistanceCategory) %>%

 group_by(Type) %>%

 mutate(Percentage = 100∗n/sum(n)) %>% ungroup()

> p_tss <- ggplot(dmrs_tss, aes(x = DistanceCategory, y = Percentage, fill = DistanceCategory)) +

 geom_col() +

 coord_flip() +

 facet_wrap(∼Type, ncol = 1) +

 labs(title = “DMR distance to nearest TSS”, x = "", y = “Percentage (%)") +

 theme_minimal(base_size = 12) +

 theme(legend.position = “right")

> ggsave(file.path(out_fig_dir, “DMR_distance_to_TSS.pdf”), p_tss, width = 7, height = 6)

20. Visualise single DMRs using DMRcate::DMR.plot() using the GRanges object generated above. Below, the dmr_granges[1] site, which corresponds to a differentially methylated site, is selected for visualisation (Figure 3).

**Figure 3:**
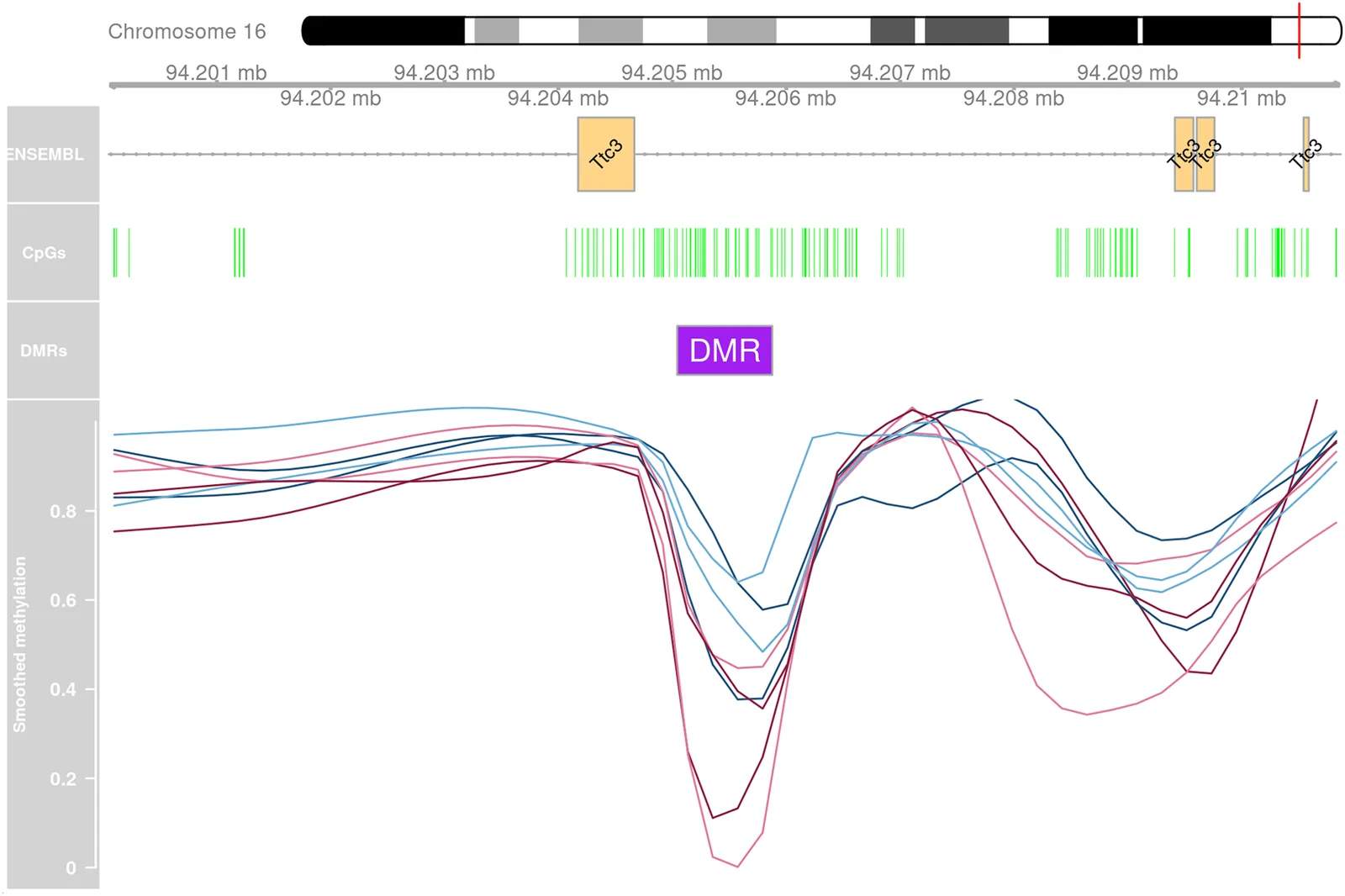
Visualisation of an example DMR in Tetratricopeptide Repeat Domain 3 (Ttc3) CpG methylation levels across the selected DMR are shown for individual samples, grouped by genotype and sex: WT-M (dark blue), WT-F (light blue), App^NL-G-F^-M (dark red), and App^NL-G-F^-F (light red). Smoothed group means and gene annotations illustrate reduced methylation across the region in the App^NL-G-F^ group.

> library(DMRcate)

> samps_all <- sampleNames(BSobj_filtered_by_regions)

*# build group labels from SampleID text (AppNL-G-F vs WT and M vs F)*

> design$Group2 <- ifelse(grepl(“ˆAppNLGF”, design$SampleID), “AppKI”, “WT")

> design$Sex2 <- ifelse(grepl(“-M-", design$SampleID), “M", “F")

> design$Grp <- paste0(design$Group2, "-", design$Sex2) # WT-M, AppKI-F.

*# reorder design to match BSseq sample order*

> design2 <- design[match(samps_all, design$SampleID), , drop = FALSE]

> stopifnot(all(design2$SampleID == samps_all))

*# assign colors*

> grp_cols <- c(

 “WT-M" = "#1F4E79″,

 “WT-F" = "#6BAED6″,

 “AppKI-M" = "#8B1E3F″,

 “AppKI-F" = "#E07A9A″

)

> phen.col <- unname(grp_cols[design2$Grp])

> stopifnot(!any(is.na(phen.col)))

> DMRcate::DMR.plot(

 ranges  = dmr_gr,

 dmr  = 1,

 CpGs   = BSobj_filtered_by_regions,

 phen.col  = phen.col,

 genome  = “mm10″

)

## Expected outcomes

The wet-laboratory protocol generates high-integrity, long-fragment genomic DNA from mouse cortex for Oxford Nanopore native DNA sequencing. Following extraction, DNA concentrations measured by Qubit typically fall within 10–100 ng/μL, with sufficient total mass available per sample for ligation-based library preparation. In this study, DNA concentrations ranged from 59.3–147.1 ng/μL, with fragment sizes of approximately 11–21 kb before standardisation (Figure 4).

**Figure 4:**
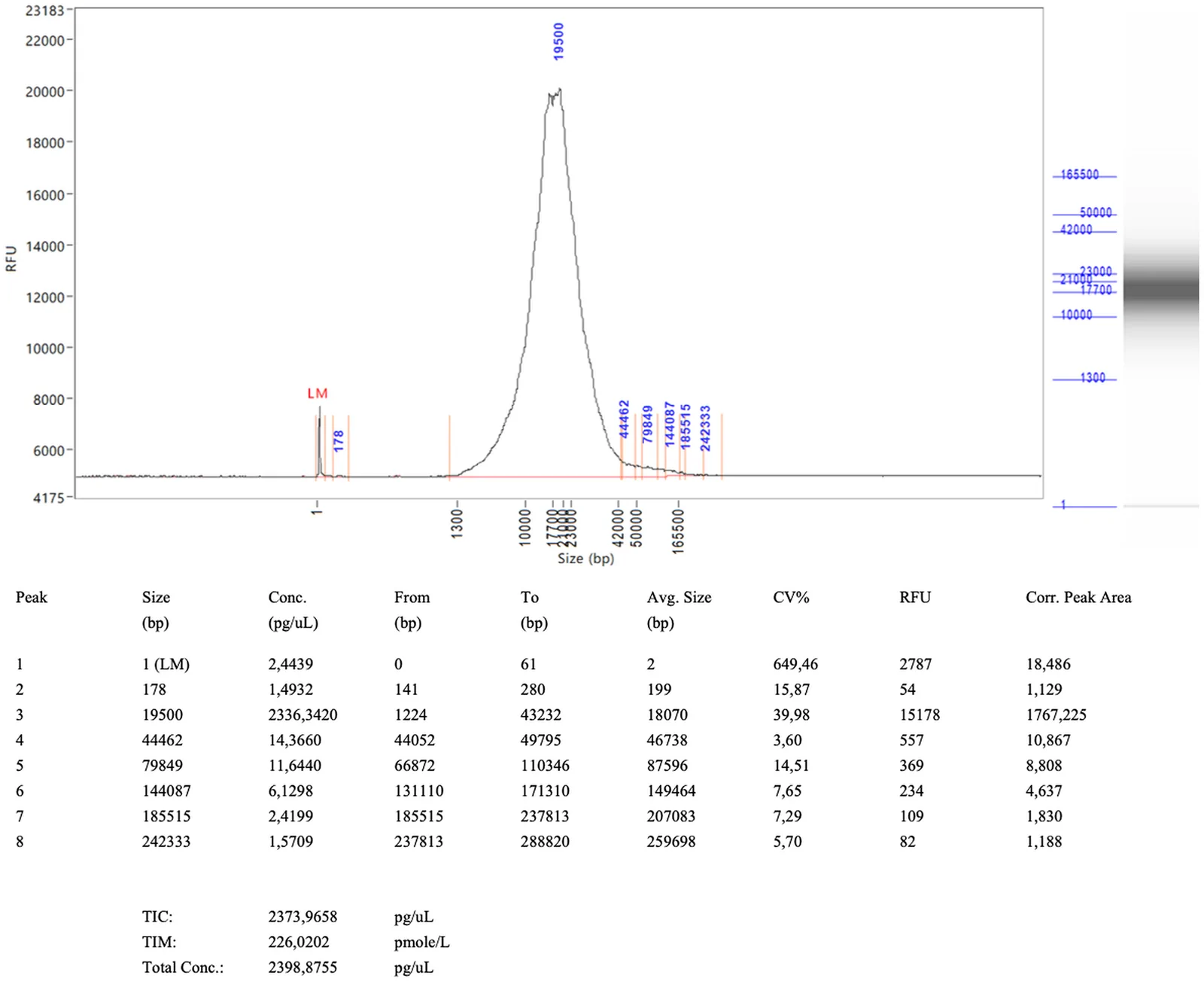
Fragment size distribution of high molecular weight genomic DNA prior to shearing Representative Femto Pulse electropherogram showing genomic DNA extracted from mouse cortex prior to mechanical shearing. The profile displays a broad high-molecular-weight distribution with a dominant peak centered at ∼19.5 kb and extended fragments reaching >100 kb, indicating high DNA integrity.

DNA was sheared using a Megaruptor to approximately 15 kb, followed by bead-based clean-up. Post-shear concentrations of representative samples were approximately 59–65 ng/μL, enabling normalization for end-repair and barcoding steps. The resulting fragment size distribution showed a dominant peak centered at 15 kb, consistent with the target library size (Figure 5). Barcoded libraries were pooled to achieve a minimum of approximately 700 ng per pool (here 701–779 ng per pool) and loaded onto R9.4.1 PromethION flow cells within ONT recommended loading range (here ∼20–32 fmol per initial load) (Table S2).

**Figure 5:**
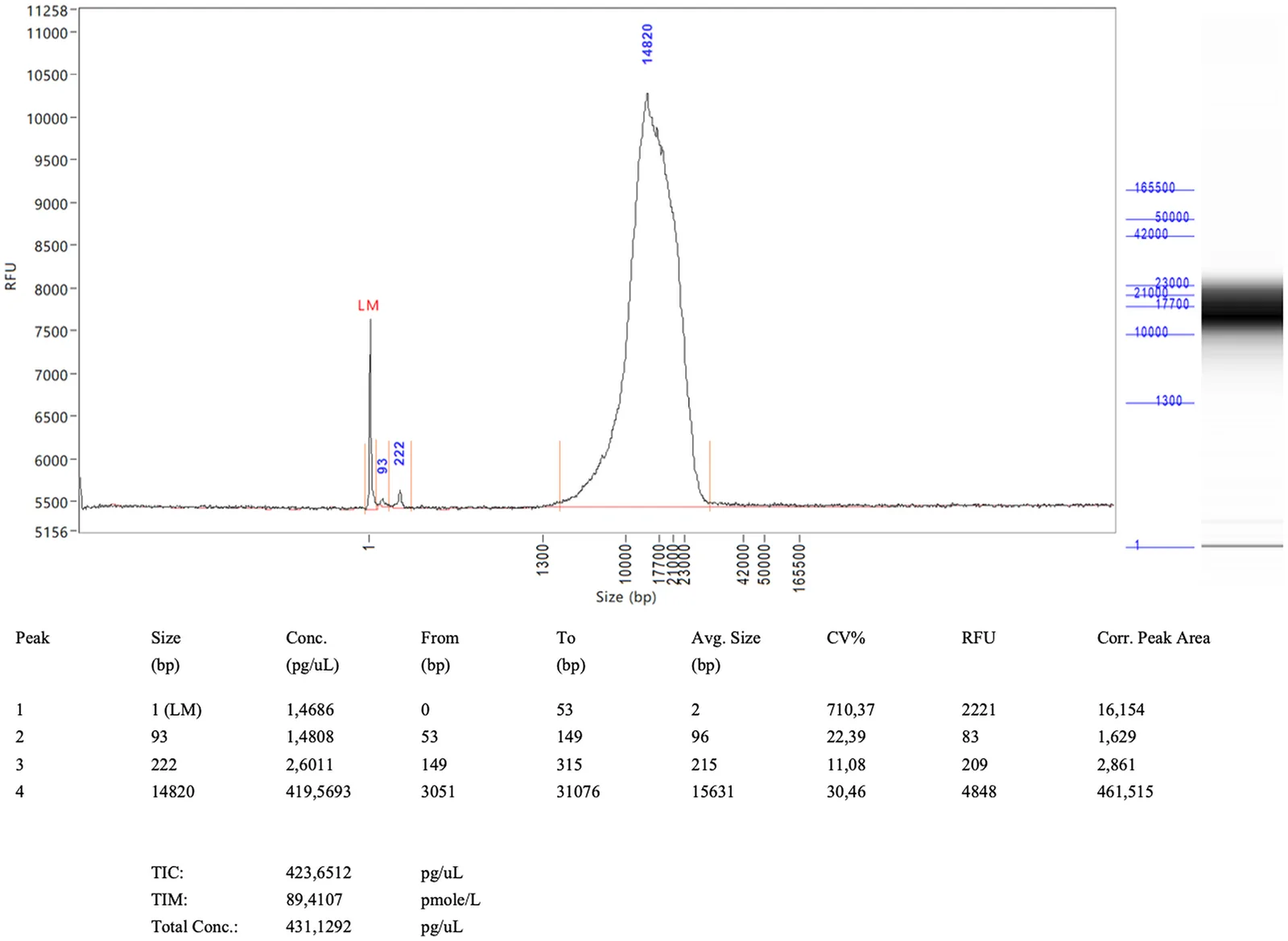
Representative Femto Pulse electropherogram showing genomic DNA after controlled shearing to ∼15 kb before ONT library preparation The profile displays a dominant fragment population centered at ∼14.8 kb (average ∼15.6 kb), with most fragments spanning ∼3–31 kb. This distribution is consistent with input requirements for long-read sequencing. Minor low-molecular-weight fragments (<300 bp) are present at low abundance.

In this study, sequencing produced 7.7–9.0 million reads per flow cell and 83.6–99.3 Gb of total yield, with read length N50 values of 12.1–18.7 kb, consistent with a ∼15 kb library target. Basecalling generated standard FASTQ and FAST5 files, as well as modBAM files containing modified base tags (MM/ML) for 5mC detection. These files are used for downstream methylation extraction and differential methylation analysis.

Two computational workflows are included in this protocol. The first workflow processes aligned, sorted, and indexed modBAM files to generate per-CpG methylation tables for each sample. These data are then combined into a BSseq object, which stores genome-wide CpG methylation measurements across all samples. In this study, the dataset contained approximately 21.86 million CpG loci across eight samples, consistent with genome-wide CpG coverage in the mouse cortex (Figure 1).

After CpG-level and region-level filtering to remove low-coverage and low-variability loci, the analysis retained millions of informative CpG sites for statistical inference. In this study, filtering retained approximately 8,513,584 CpG loci across 1,357,100 genomic regions, depending on coverage and variability thresholds.

Multi-factor DSS modeling produced per-CpG test statistics for genotype-associated methylation differences while adjusting for covariates such as sex and sequencing batch. Because multiple-testing correction across millions of loci is stringent (Table 1), interpretation typically focuses on spatially clustered CpG changes aggregated into DMRs. In this study, DMR calling identified 2,735 DMRs, of which 68 overlapped promoter regions, and 2,667 were distal regions, assigned to the nearest transcription start site for gene annotation (Table 2).

**Table 1:** Top 5 differentially methylated loci identified between App^NL-G-F^ and wild-type cortex

Chromosome	Position (bp, mm10)	Wald statistic	P-value	FDR
chr8	63001848	4.470525	7.80 × 10^−6^	1
chr19	6484382	−4.145498	3.39 × 10^−5^	1
chr13	4627796	4.143325	3.42 × 10^−5^	1
chr12	86816431	4.031889	5.53 × 10^−5^	1
chr8	63001986	4.013995	5.96 × 10^−5^	1

False discovery rate (FDR) values were close to ∼1 for all loci. This is expected when applying Benjamini–Hochberg correction across a very large number of tests (∼8.5 million CpG sites). Even relatively small p values (e.g. ∼10^–5^) may not remain significant after multiple testing correction at this scale. Clusters of CpG sites with consistent signal (e.g., adjacent loci on chr8) likely represent true localised methylation change, despite lack of single-site FDR significance. Therefore, biological interpretation should not rely solely on individual CpG significance.

**Table 2:** Annotated differentially methylated regions identified between App^NL-G-F^ and wild-type cortex

Chromosome	Start (bp)	End (bp)	Area statistics	Genomic annotation	Nearest gene	Distance to TSS (bp)
chr12	3235412	3236009	−118.19	Promoter (−2000,+500)	Rab10os	0
chr2	116061381	116062065	79.35	Distal (nearest TSS)	G630016G05Rik	5159
chr6	4748102	4748493	77.73	Promoter (−2000,+500)	Sgce	0
chr2	118554994	118555470	76.77	Distal (nearest TSS)	Bmf	5306
chr11	51668675	51669189	−71.09	Distal (nearest TSS)	D930048N14Rik	17720

This table includes DMR genomic coordinates (chr), areaStat, promoter/distal annotation (annotation), nearest gene assignment as gene symbol, and distance to the nearest transcription start site (distance to TSS).

Overall, the observed sequencing throughput and read-length distribution are consistent with published benchmarks for ONT PromethION sequencing using R9.4.1 flow cells and ligation-based libraries. In a large-scale comparative study using primarily R9.4 chemistry, accurate genome-wide CpG methylation detection was achieved between 10–12× coverage and improved above 20×, with typical read N50 values in the range of 10–20 kb.^40^ Although the newer R10.4 chemistry provides improved base-calling accuracy and reduced strand bias, the performance characteristics of our dataset fall within the expected range for stable genome-wide CpG coverage suitable for robust differential methylation analysis.

## Limitations

This workflow focuses on CpG 5mC and does not distinguish other modifications, and so is not designed to annotate others for the downstream analysis, although the pipeline can be used to generate tables to infer statistical DMR/DML changes for other modifications too. DMR-to-gene assignment by nearest gene/TSS is imperfect, especially for distal regulatory regions. Bulk cortical DNA (minimum 1 μg sheared DNA) reflects a mixture of cell types; cell-type enrichment is indirect without cell-sorted methylomes. Genome-wide analysis is computationally intensive, particularly for large per-site objects and multi-factor modeling.

## Troubleshooting

### Problem 1

Low sequencing yield or insufficient CpG coverage.

**Related to:** *Extract high-molecular-weight genomic DNA from cortex*; *Prepare ONT native barcoded ligation libraries*; and *Run PromethION sequencing and perform 5mC-enabled basecalling*

### Possible cause

Low CpG coverage is most caused by degraded or fragmented genomic DNA, insufficient DNA input during library preparation, excessive multiplexing per flow cell, or reduced sequencing performance. These factors reduce the number of reads covering each CpG site and limit statistical power for downstream differential methylation analysis.

### Solution

The selection of a genomic DNA extraction kit is crucial. One example is mentioned in this protocol; it should be suitable for obtaining high-molecular-weight material (>50 kb), and the DNA should be handled gently to avoid fragmentation. Avoid vortexing, repeated freeze–thaw cycles, excessive pipetting, and use wide-bore pipettes. Use at least 1 μg sheared DNA per sample for library preparation. If coverage is insufficient, reduce the number of multiplexed samples per flow cell to increase sequencing depth per sample. For genome-wide methylation analysis in mice, sequencing depths of approximately 10–15× genome coverage per sample are generally sufficient for stable CpG detection. This corresponds to 7.7–9.0 million reads with read length N50 values of 12.1–18.7 kb, and 83.6–99.3 Gb yield per flow cell, which was distributed across 2 multiplexed samples. Filtering thresholds should be optimized empirically based on CpG retention and coverage distributions.

### Problem 2

Modified-base tags (MM/ML) are missing in the BAM file

**Related to:** *Run PromethION sequencing and perform 5mC-enabled basecalling*; and *Read alignment and methylation extraction.*

### Possible cause

Modified-base detection may not have been enabled during basecalling, or modification tags may have been lost during BAM processing, FASTQ conversion, or realignment steps. Without these tags, methylation information cannot be recovered.

### Solution

Basecalling should be performed using a modified-base-enabled Guppy model with 5mC detection enabled. When converting BAM files to FASTQ before alignment, preserve modification tags using appropriate samtools options. After alignment and sorting, inspect BAM files to confirm the presence of MM and ML tags. If tags are absent, regenerate modBAM files from the original basecalling output with modified-base detection enabled.

### Problem 3

DSS model fitting fails or produces errors related to insufficient degrees of freedom

**Related to:** *Genotype animals and define the experimental design*; *Data import and initial preprocessing*; and *Differential methylation analysis*

### Possible cause

This typically occurs when the statistical model contains too many covariates relative to the number of samples, or when experimental variables such as genotype and batch are confounded due to unbalanced experimental design.

### Solution

The samples should be balanced across biological and technical variables such as genotype, sex, and sequencing pool. Include sequencing pool or flow cell as a batch covariate where appropriate. If replication is limited, simplify the statistical model by removing interaction terms or unnecessary covariates. Verify that the design matrix is of full rank before fitting the DSS model.

## Resource availability

### Lead contact

Requests for additional information and resources should be directed to the lead contact, Dervis A. Salih (dervis.salih@ucl.ac.uk).

### Technical contact

Requests for further information and technical details about the protocol should be directed to Ümran Yaman (umran.yaman.19@ucl.ac.uk).

### Materials availability

This study did not generate new unique reagents.

### Data and code availability

• All original code has been deposited in Zenodo and is publicly available. Relevant DOIs and GitHub repository links are provided in the key resources table.
• Any additional information required to reanalyze the data reported in this study is available from the lead contact upon reasonable request.

## Acknowledgments

We would like to deeply thank Gareth Banks, Rasneer Sonia Bains, Hamish Forrest, Michelle Stewart, Tatiana V. Lipina, and Sara E. Wells at the MRC Mary Lyon Centre for their great help and support with mouse husbandry, genotyping, phenotyping, and technical assistance. U.Y. is employed in J.H.’s laboratory with support from the Fidelity Bermuda Foundation. D.A.S. and J.H. are supported by the 10.13039/501100017510UK Dementia Research Institute (award no. UKDRI-1210) through UK DRI Ltd., principally funded by the 10.13039/501100000265UK Medical Research Council (MRC). D.A.S. also received funding from an MRC award MC_PC_23036 to establish an MRC National Mouse Genetics Network Ageing Cluster. D.A.S. and J.H. are additionally supported by the Dolby Foundation. J.H. is also supported by the 10.13039/501100012621NIHR University College London Hospitals Biomedical Research Centre. F.W. and P.M. are supported by the 10.13039/501100017510UK Dementia Research Institute (UKDRI-1014 and UKDRI-CIP0202 held by F.W.) through 10.13039/501100017510UK DRI Ltd., principally funded by the 10.13039/501100000265UK Medical Research Council. F.W. was also supported by an Alzheimer’s Research UK Senior Research Fellowship (ARUK-SRF2018A-001).

## Author contributions

U.Y. designed the study, performed the experiments and analyses, and wrote and edited the manuscript. E.G. contributed to the analysis and edited the manuscript. F.W., P.M., and J.H. contributed to the experimental design and edited the manuscript; F.W. also obtained ethical approval. P.M. conducted the *in vivo* research. J.L. and H.U. performed library preparation, quality control, and sequencing; H.U. also edited the manuscript. D.A.S. co-designed the study, contributed to the analysis, and wrote and edited the manuscript.

## Declaration of interests

D.A.S. and J.H. kindly received funding for a non-overlapping project from Eli Lilly. J.H. received consultancy money from Eisai. F.W. has undertaken for fee consultancy for Alnylam Pharmaceuticals and TRIMTECH THERAPEUTICS for work unconnected to this manuscript; she also sits on the Science Advisory Board for Cambridge Phenotyping and is a member of the Executive Board of the MRC National Mouse Genetics Network and the Trisomy 21 Research Society.

## Supplemental information

Document S1. Figure S1

Table S1. Primer and probe sequences used for TaqMan-based genotyping of the App^NL-G-F^ mouse model, related to Step 8

Table S2. DNA input, library preparation, and sequencing metrics for Oxford Nanopore PromethION runs, related to Step 24 and Expected Outcomes

## References

[1] Gu Z., Huebschmann D. (2022). rGREAT: An R/Bioconductor package for functional enrichment on genomic regions. Bioinformatics.

[2] Gu Z., Huebschmann D. (2022). simplifyEnrichment: An R/Bioconductor package for clustering and visualizing functional enrichment results. Genom. Proteom. Bioinform..

[3] New England Biolabs. Protocol for Genomic DNA Cleanup (T3010). New England Biolabs.

[4] Dahl C., Grønbæk K., Guldberg P. (2011). Advances in DNA methylation: 5-hydroxymethylcytosine revisited. Clin. Chim. Acta.

[5] Hon G.C., Song C.X., Du T., Jin F., Selvaraj S., Lee A.Y., Yen C.A., Ye Z., Mao S.Q., Wang B.A. (2014). 5mC oxidation by Tet2 modulates enhancer activity and timing of transcriptome reprogramming during differentiation. Mol. Cell.

[6] Du Q., Luu P.L., Stirzaker C., Clark S.J. (2015). Methyl-CpG-binding domain proteins: Readers of the epigenome. Epigenomics.

[7] Jones P.A. (2012). Functions of DNA methylation: Islands, start sites, gene bodies and beyond. Nat. Rev. Genet..

[8] Ni P., Nie F., Zhong Z., Xu J., Huang N., Zhang J., Zhao H., Zou Y., Huang Y., Li J. (2023). DNA 5-methylcytosine detection and methylation phasing using PacBio circular consensus sequencing. Nat. Commun..

[9] Bibikova M., Le J., Barnes B., Saedinia-Melnyk S., Zhou L., Shen R., Gunderson K.L. (2009). Genome-wide DNA methylation profiling using Infinium® assay. Epigenomics.

[10] Bibikova M., Barnes B., Tsan C., Ho V., Klotzle B., Le J.M., Delano D., Zhang L., Schroth G.P., Gunderson K.L. (2011). High density DNA methylation array with single CpG site resolution. Genomics.

[11] Harris R.A., Wang T., Coarfa C., Nagarajan R.P., Hong C., Downey S.L., Johnson B.E., Fouse S.D., Delaney A., Zhao Y. (2010). Comparison of sequencing-based methods to profile DNA methylation and identification of monoallelic epigenetic modifications. Nat. Biotechnol..

[12] Laird P.W. (2010). Principles and challenges of genomewide DNA methylation analysis. Nat. Rev. Genet..

[13] Logsdon G.A., Vollger M.R., Eichler E.E. (2020). Long-read human genome sequencing and its applications. Nat. Rev. Genet..

[14] Wang Y., Zhao Y., Bollas A., Wang Y., Au K.F. (2021). Nanopore sequencing technology, bioinformatics and applications. Nat. Biotechnol..

[15] Ebbert M.T.W., Jensen T.D., Jansen-West K., Sens J.P., Reddy J.S., Ridge P.G., Kauwe J.S.K., Belzil V., Pregent L., Carrasquillo M.M. (2019). Systematic analysis of dark and camouflaged genes reveals disease-relevant genes hiding in plain sight. Genome Biol..

[16] Kunkle B.W., Grenier-Boley B., Sims R., Bis J.C., Damotte V., Naj A.C., Boland A., Vronskaya M., van der Lee S.J., Amlie-Wolf A. (2019). Genetic meta-analysis of diagnosed Alzheimer’s disease identifies new risk loci and implicates Aβ, tau, immunity and lipid processing. Nat. Genet..

[17] Lambert J.C., Ibrahim-Verbaas C.A., Harold D., Naj A.C., Sims R., Bellenguez C., DeStafano A.L., Bis J.C., Beecham G.W., Grenier-Boley B. (2013). Meta-analysis of 74,046 individuals identifies 11 new susceptibility loci for Alzheimer’s disease. Nat. Genet..

[18] Bellenguez C., Küçükali F., Jansen I.E., Kleineidam L., Moreno-Grau S., Amin N., Naj A.C., Campos-Martin R., Grenier-Boley B., Andrade V. (2022). New insights into the genetic etiology of Alzheimer’s disease and related dementias. Nat. Genet..

[19] Graham A.C., Bellou E., Harwood J.C., Yaman U., Celikag M., Magusali N., Rambarack N., Botia J.A., Sala Frigerio C., Hardy J. (2025). Human longevity and Alzheimer’s disease variants act via microglia and oligodendrocyte gene networks. Brain.

[20] De Jager P.L., Srivastava G., Lunnon K., Burgess J., Schalkwyk L.C., Yu L., Eaton M.L., Keenan B.T., Ernst J., McCabe C. (2014). Alzheimer’s disease: Early alterations in brain DNA methylation at ANK1, BIN1, RHBDF2 and other loci. Nat. Neurosci..

[21] Lardenoije R., Roubroeks J.A.Y., Pishva E., Leber M., Wagner H., Iatrou A., Smith A.R., Smith R.G., Eijssen L.M.T., Kleineidam L. (2019). Alzheimer’s disease-associated (hydroxy)methylomic changes in the brain and blood. Clin. Epigenet..

[22] Marzi S.J., Leung S.K., Ribarska T., Hannon E., Smith A.R., Pishva E., Poschmann J., Moore K., Troakes C., Al-Sarraj S. (2018). A histone acetylome-wide association study of Alzheimer’s disease identifies disease-associated H3K27ac differences in the entorhinal cortex. Nat. Neurosci..

[23] Fodder K., Smith H.M.G., Yaman U., Piras I.S., Murthy M., Hardy J., Lashley T., de Silva R., Salih D.A., Bettencourt C. (2026). Early oligodendrocyte dysfunction signature in Alzheimer’s disease: Insights from DNA methylomics and transcriptomics. Mol. Psychiatry.

[24] Saito T., Matsuba Y., Mihira N., Takano J., Nilsson P., Itohara S., Iwata N., Saido T.C. (2014). Single App knock-in mouse models of Alzheimer’s disease. Nat. Neurosci..

[25] Benitez D.P., Jiang S., Wood J., Wang R., Hall C.M., Peerboom C., Wong N., Stringer K.M., Vitanova K.S., Smith V.C. (2021). Knock-in models related to Alzheimer’s disease: synaptic transmission, plaques and the role of microglia. Mol. Neurodegener..

[26] Peters T.J., Buckley M.J., Chen Y., Smyth G.K., Goodnow C.C., Clark S.J. (2021). Calling differentially methylated regions from whole genome bisulphite sequencing with DMRcate. Nucleic Acids Res..

[27] Feng H., Conneely K.N., Wu H. (2014). A Bayesian hierarchical model to detect differentially methylated loci from single nucleotide resolution sequencing data. Nucleic Acids Res..

[28] Wu H., Wang C., Wu Z. (2013). A new shrinkage estimator for dispersion improves differential expression detection in RNA-seq data. Biostatistics.

[29] Wu H., Xu T., Feng H., Chen L., Li B., Yao B., Qin Z., Jin P., Conneely K.N. (2015). Detection of differentially methylated regions from whole-genome bisulfite sequencing data without replicates. Nucleic Acids Res..

[30] Park Y., Wu H. (2016). Differential methylation analysis for BS-seq data under general experimental design. Bioinformatics.

[31] Li H. (2018). Minimap2: Pairwise alignment for nucleotide sequences. Bioinformatics.

[32] Mölder F., Jablonski K.P., Letcher B., Hall M.B., Tomkins-Tinch C.H., Sochat V., Forster J., Lee S., Twardziok S.O., Kanitz A. (2021). Sustainable data analysis with Snakemake. F1000Res..

[33] Lawrence M., Huber W., Pagès H., Aboyoun P., Carlson M., Gentleman R., Morgan M.T., Carey V.J. (2013). Software for computing and annotating genomic ranges. PLoS Comput. Biol..

[34] Hansen K.D., Langmead B., Irizarry R.A. (2012). BSmooth: From whole genome bisulfite sequencing reads to differentially methylated regions. Genome Biol..

[35] Mordaunt C.E., Mouat J.S., Schmidt R.J., LaSalle J.M. (2022). Comethyl: A network-based methylome approach to investigate the multivariate nature of health and disease. Briefings Bioinf..

[36] Morgan M., Pagès H., Obenchain V., Hayden N. (2016). Rsamtools: Binary alignment (BAM), FASTA, variant call (BCF), and tabix file import. R package version 2.28.0. https://bioconductor.org/packages/Rsamtools/.

[37] Wickham H. (2016). https://ggplot2.tidyverse.org.

[38] Wickham H., François R., Henry L., Müller K., Vaughan D. (2026). dplyr: A grammar of data manipulation. R package version 1.2.1. https://dplyr.tidyverse.org.

[39] Federation of European Neuroscience Societies (FENS) Isolation of adult mouse hippocampus. https://www.youtube.com/watch?v=XUbIOG1Euf4.

[40] Sigurpalsdottir B.D., Stefansson O.A., Holley G., Beyter D., Zink F., Hardarson M.þ., Sverrisson S.þ., Kristinsdottir N., Magnusdottir D.N., Magnusson O.þ. (2024). A comparison of methods for detecting DNA methylation from long-read sequencing of human genomes. Genome Biol..

