# Supplementary material for: Protocol for genome-wide differential methylation using long-read DNA sequencing in the cortex of a mouse model of Alzheimer’s disease
Source: STAR Protoc. 2026 Oct 8;7(4):104827. doi: 10.1016/j.xpro.2026.104827 (PMC13675703; doi:10.1016/j.xpro.2026.104827)
Supplement: Supplementary file 1 [file mmc1.pdf]

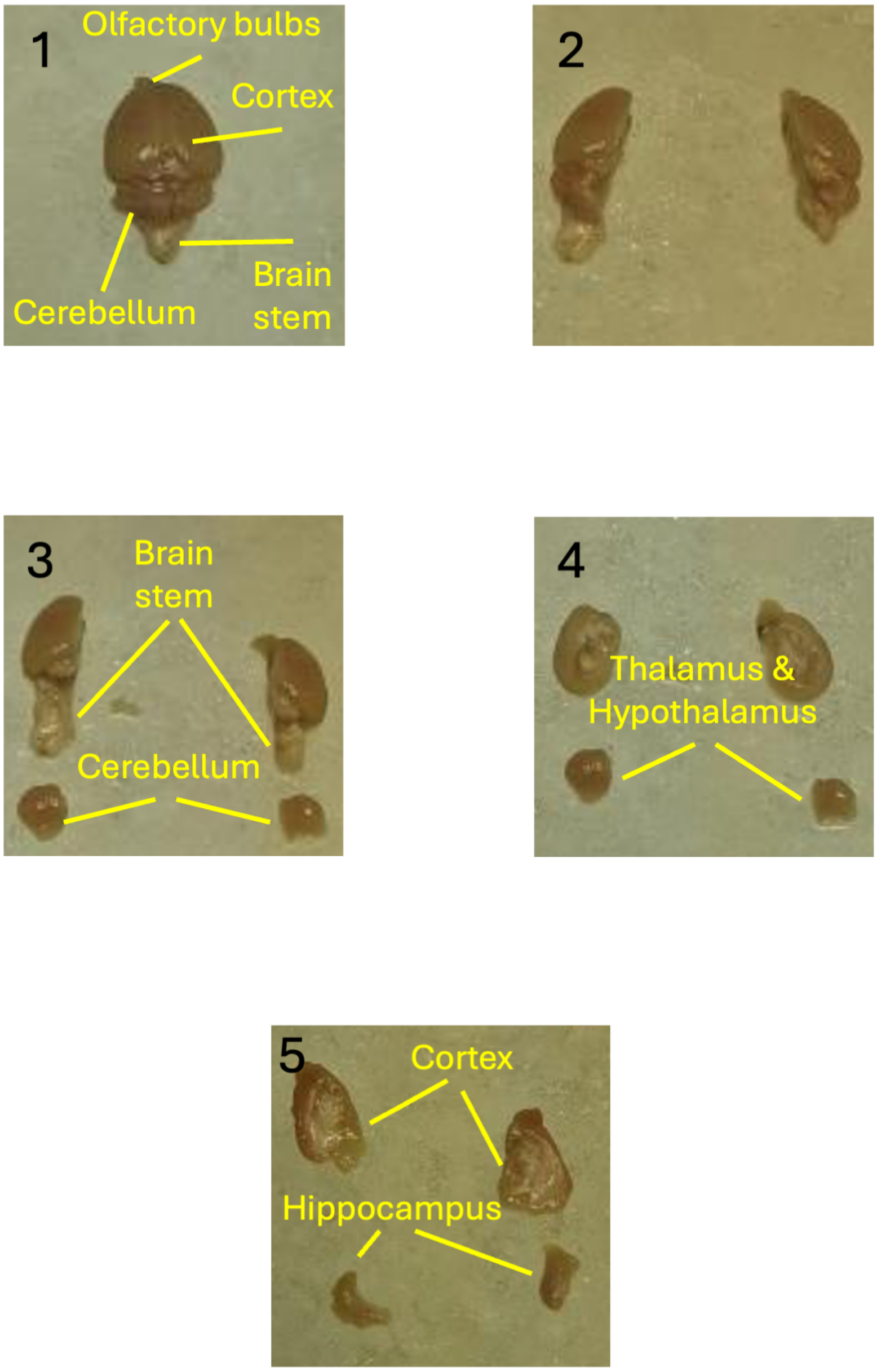

Supplementary Figure 1. Dissection of the murine cortex.
(1) Whole brain was extracted from the skull and oriented to visualise the olfactory bulbs, cerebral cortex, cerebellum, and brainstem. (2) The brain was bisected along the midline into two hemispheres. (3) The cerebellum was removed from each hemisphere. (4) Subcortical structures, including the brainstem, midbrain, thalamus, and hypothalamus, were dissected away to isolate the cortical mantle. (5) The hippocampus was carefully dissected from the cortical tissue using blunt dissection, and the olfactory bulbs were excised. The left and right cortical hemispheres were then individually snap-frozen on dry ice.
